# Coopetition, exploration and exploitation capabilities, and growth performance in digital healthcare ventures

**DOI:** 10.3389/fpubh.2024.1369885

**Published:** 2024-06-19

**Authors:** Xiangmin Liu, Li Bai, Xiaoning Leng, Yexiang Yao, Yue Yang, Debao Li, Haobo Yin

**Affiliations:** ^1^Personal Department, Qiqihar Medical University, Qiqihar, China; ^2^School of Public Health, Qiqihar Medical University, Qiqihar, China; ^3^Medical Affairs Department, Qiqihar Medical University, Qiqihar, China

**Keywords:** coopetition, exploration and exploitation capabilities, growth performance, environmental uncertainty, digital healthcare ventures

## Abstract

**Introduction:**

Studies focusing on coopetition and dynamic capabilities have expanded significantly over the past several decades. Coopetition strategy and dynamic capabilities are increasingly recognised as sources of sustained competitive advantage. The purpose of this paper is to provide a better understanding of the factors driving growth performance in digital healthcare ventures by examining the role of coopetition, exploration and exploitation capabilities, and environmental uncertainty. While numerous studies have examined the competitive advantage of coopetition, its specific contribution to the growth of ventures in the digital realm remains less explored. Clarifying the strategic role of coopetition in driving growth performance is critical for delineating the intricate relationship between coopetition and growth performance, particularly in the context of digital healthcare ventures. To fill in this research gap, this study uses coopetition theory and dynamic capabilities theory to look at how exploration and exploitation capabilities, as well as environmental uncertainty, affect the relationship between coopetition and growth performance in digital healthcare ventures.

**Methods:**

We collected a total of 338 questionnaires from Chinese digital healthcare ventures between March 2023 and August 2023. We conducted data analysis using SPSS 26.0 and its macro-program PROCESS.

**Results:**

Our results confirm that coopetition has a positive effect on growth performance in digital healthcare ventures. Furthermore, exploration and exploitation capabilities fully mediate the relationship between coopetition and growth performance. Moreover, environmental uncertainty significantly and distinctively moderates the impact of exploration and exploitation capabilities on growth performance.

**Discussion:**

This study contributes to the existing literature by providing deeper insight into the relationship between coopetition and growth performance in digital healthcare ventures. It also offers important practical implications for public health improvement and socio-economic development.

## 1 Introduction

Historically, big tech companies have made substantial contributions to healthcare, while the impact of digital healthcare ventures has often been underappreciated and overlooked. Recently, these ventures have seen a significant surge in growth, capturing the attention of policymakers, investors, entrepreneurs, and healthcare professionals alike (1, 2). The COVID-19 pandemic acted as a catalyst for this transformation, highlighting the essential role of digital innovations in public healthcare (3, 4). Digital healthcare ventures help solve social problems such as the uneven distribution of healthcare resources and tensions between medical professionals and patients (5). Despite their growing prevalence, there remains a relative lack of understanding about the development of this sector (1). In reality, balancing profitability with the public welfare of healthcare services remains a challenge for many digital healthcare ventures, alongside implementation difficulties and concerns over profitability and innovation pathways (6). Despite opportunities for growth during the COVID-19 pandemic (3), the sustainability of these ventures post-pandemic remains uncertain. To address the issues raised above, this study attempts to explore the antecedents and mechanisms of venture growth in digital healthcare.

Technologies such as artificial intelligence (AI), machine learning (ML), the Internet of Things (IoT), virtual reality (VR), and blockchain are revolutionizing business models and value creation in the target healthcare market (7, 8). This digital transformation is not only vital for improving health conditions but is also crucial for the well-being of humanity (7). Countries such as Ireland and France are fostering partnerships with digital health companies, helping shape and influence national health, and striving to become global leaders in digital public health (9, 10). Digital healthcare ventures provide substantial technical and data support for public health management (8), extending beyond mere technology-enabled healthcare services or online consultation platforms to enhance the accuracy, efficiency, and responsiveness of healthcare systems to public health emergencies (1). Furthermore, these ventures play a critical role in not only gradually achieving a digitalisation-driven upgrade of the healthcare industry, but also promoting health equity in public health by delivering cost-effective solutions that enhance digital health literacy and provide individualised care (1, 11). Therefore, this study is invaluable for improving public health outcomes and individual well-being.

The strategy of coopetition, which blends cooperation and competition, has been widely recognised as a potent strategy for firms seeking superior performance (12–15). This approach allows firms to interact with various players in the value network, including suppliers, customers, competitors, and complementors, to gain a competitive advantage or value (16–18). To contribute to the development of coopetition research, it is necessary to empirically examine the consequences of coopetition in different contexts. While previous research on coopetition has primarily focused on large or mature firms as a theoretical assumption (19, 20), digital healthcare ventures operate in a markedly different environment, facing unique market dynamics and competitive strategies (5, 8). According to the coopetition theory, inter-organisational collaborations contribute to the scale and innovation advantage of entrepreneurial ventures and facilitate their profound growth (21, 22). However, previous studies have not identified whether and how coopetition contributes to growth performance in digital healthcare ventures (23–25). Therefore, addressing this gap is crucial for understanding how coopetition can better support these ventures in achieving enhanced growth performance (26).

Following the research stream on coopetition, it is important to provide insights into the underlying mechanisms through which coopetition can drive growth performance in digital healthcare ventures. According to the dynamic capabilities theory, it is suggested that coopetition strategies could harness ambidextrous capabilities to effectively manage and reconfigure knowledge and resources within the digital environment, thereby enhancing growth (27). The ambidextrous capabilities paradigm enables organisations to maintain a competitive edge by adeptly balancing two complementary processes: exploration and exploitation. Exploration involves activities such as search, variation, risk-taking, experimentation, flexibility, and discovery, while exploitation focuses on refinement, choice, production, efficiency, selection, implementation, and execution (28). These capabilities not only allow an organisation to adapt swiftly to environmental changes but also optimise the use of existing resources to enhance functions like R&D and marketing. Although firms implementing coopetition strategies can leverage relationships with different stakeholders to achieve growth (29), they have to decide “how to allocate the knowledge and resources” for long-term and short-term development. While previous studies have focused on the relationship between firm growth and the benefits that coopetition brings, there is a relative gap in understanding how ambidextrous capabilities function as a mediating link within this relationship and where the causality begins (30–32). Therefore, there is a need to focus on the mediating role of ambidextrous capabilities in the relationship between coopetition and growth performance in digital healthcare ventures.

Environmental uncertainty is attracting practitioners and policymakers’ attention as an environmental factor affecting firm growth (33–35). Environmental uncertainty, which refers to the rate and intensity of technological and market changes, plays a pivotal role in the growth of digital healthcare ventures (36). Complementary knowledge and resources are essential for a firm to grow; however, these assets are highly susceptible to external environmental variables (37). Consequently, environmental uncertainty exerts a contingent influence on the mechanism of action dominated by internal variables, especially in the context of inter-organisational collaboration (33). Although previous studies have established a strong relationship between organisational ambidexterity and firm performance, considering environmental conditions (38–41), there is still a lack of adequate explanations on whether and how external environmental factors affect venture growth in digital healthcare. Therefore, this paper seeks to examine the contingent influence of environmental uncertainty on the relationship between ambidextrous capabilities and growth performance in digital healthcare ventures. The research questions are as follows: (1) Do exploration and exploitation capabilities play mediating roles in the influence of coopetition on growth performance in digital healthcare ventures? (2) Under environmental uncertainty, how do digital healthcare ventures improve growth performance through exploration and exploitation capabilities? In order to answer the questions, this paper carries out rigorous empirical research based on the cross-sectional survey data collected from digital healthcare ventures.

By addressing the research questions outlined above, this study has the potential to contribute theoretically in three aspects: Firstly, it advances the coopetition theory by incorporating digital healthcare scenarios in the analysis of the relationship between coopetition and venture growth. Secondly, this study expands theoretical research on ambidextrous capabilities by uncovering the mediating roles of exploration and exploitation capabilities. This sheds light on the intricate relationship between coopetition and growth performance in digital healthcare ventures, thereby facilitating the integration of coopetition theory and dynamic capability theory within the digital healthcare context. Thirdly, this study enriches the understanding of the boundaries of ambidextrous capabilities in digital contexts, clarifying the differential roles of exploration and exploitation capabilities for the growth performance in digital healthcare ventures under environmental uncertainty.

## 2 Theoretical background and hypotheses

### 2.1 Coopetition theory

Brandenburger and Nalebuff (42) first introduced the concept of coopetition, a business strategy that blends competition and cooperation among enterprises, suppliers, customers, and other value network actors (12). Subsequent scholarly analyses, rooted in game theory, have characterised coopetition as a plus-sum game, diverging from the zero-sum framework and suggesting potential for mutually beneficial outcomes (43). This perspective posits that profits for one party need not come at the expense of the other, fostering win-win possibilities (42, 44). Academic exploration of coopetition has described it as a dual dynamic wherein firms engage in both competition and cooperation (19, 21, 45). Alternatively, it has been viewed through tripartite relationships among affiliated enterprises (46, 47), or as a characteristic of a broader business network, where multiple enterprises engage in both competitive and cooperative interactions (13, 45). Kennedy et al. (48) asserts that firms are more effective at sharing and combining resources with other participants to create value (or performance) than when acting alone to achieve competitive advantage. Similarily, Wu et al. (49) argue that, in a dynamic and evolving business ecosystem, firms can create and capture new value through more complex patterns of cooperation and competition. In the contemporary business landscape, driven by digitalisation in the fourth industrial revolution, competition and cooperation among companies are increasingly intertwined (24). In the digital economy era, advancements in digital technology have reshaped business models and societal norms, thereby intensifying coopetition within the healthcare sector (3). As traditional business models prove inadequate for contemporary demands, coopetition becomes a strategic imperative for digital healthcare ventures (23). The ongoing digitalisation wave compels these ventures to actively engage and partner with stakeholders, including similar firms, hospitals and healthcare institutions, drug or device manufacturers, distributors, financial and insurance institutions, and healthcare administrations, and other relevant parties (50, 51). Drawing from the existing research and considering the context of digital healthcare, this paper posits that coopetition is a strategic approach whereby these entities seek competitive advantage or value through collaboration with a diverse array of participants in the value network. This includes engagement with fellow digital healthcare companies, healthcare organisations, medical professionals, patients, equipment manufacturers, logistics firms, academic institutions, local government bodies, and other relevant stakeholders (14, 52, 53).

### 2.2 Dynamic capabilities theory

The dynamic capabilities theory serves as the cornerstone of this study. At its core, this theory advocates for firms to adeptly integrate and reconfigure internal and external resources in response to rapidly changing external conditions, building core capabilities for competitive advantage (54, 55). In the evolving digital landscape, the traditional approach of enhancing competitiveness through resource acquisition, such as capital, labor, and raw materials, is gradually losing relevance across many industrial sectors due to the limitations of the “diminishing returns to scale” model in the global business environment (24). As digital technology blurs the boundaries between actors and the complexity of the business environment increases, the importance of dynamic capabilities becomes even more pronounced (56). Teece (57) argues that in complex and rapidly changing business ecosystems, understanding the significance of achieving competitive advantage hinges on dynamic capability perspectives. Therefore, considering the enhancement of growth performance through dynamic capabilities is imperative. Dynamic capabilities are crucial in driving growth in the digital healthcare sector, offering a means to navigate the complexities of the digital landscape (58). It is crucial to identify the dynamic capabilities associated with digitisation, as they frequently serve as sources of sustained competitive advantage. Central to the dynamic capabilities perspective is the notion of ambidexterity—an essential organisational attribute and firm capability. The theory posits that a firm’s fundamental capabilities should not only secure short-term competitive positions but also evolve into long-term competitive advantages (55). We propose, drawing upon the characteristics of the dynamic capabilities perspective, that firms can view exploration and exploitation capabilities as dynamic capabilities, enabling them to succeed in fluctuating and uncertain business climates (58). Hence, digital healthcare ventures must enhance their exploration and exploitation capabilities to address current challenges and foster future growth.

The concept of “ambidexterity” was first introduced to management academia by Duncan (59), describing organisational capabilities. March (28) later defined organisational learning ambidexterity in terms of exploration and exploitation. Organisations aim to balance or combine exploratory and exploitative learning modes to achieve efficiency and innovation (60). In the digital age, exploration and exploitation capabilities have become essential for the growth of digital healthcare ventures (61). This study posits that firms focusing solely on exploitative (incremental) or exploratory (radical) innovation cannot thrive amidst fierce competition (55, 62). While exploration capabilities enable ventures to overcome organisational inertia, lacking exploitation capabilities may result in a failure trap where creativity fails to translate into practical improvement. Conversely, ventures emphasizing exploitation might fall into a capability trap, becoming overly reliant on past successes and struggling to remain competitive in a rapidly changing digital world (27). Thus, these capabilities are complementary and interlinked. In the digital economy, characterised by increasingly unstable and intricate external environments, ambidextrous capabilities, which balance long-term and short-term benefits, have garnered significant academic interest (61). These dynamic capabilities enable an organisation to both explore and exploit within dynamic settings, with each capability type serving distinct purposes, functions, knowledge bases, implementation paths, and yielding different innovation outcomes. First, exploration capabilities focus on creating new markets and user needs, while exploitation capabilities aim to satisfy existing users and address current market challenges. Second, exploration introduces new products, services, technologies, and processes to seek competitive advantage, while exploitation improves existing ones to enhance operational efficiency. Third, exploration relies on new knowledge and skills, whereas exploitation builds on and updates existing knowledge. Fourth, exploration encourages discovering opportunities through innovative activities, while exploitation stresses refining and enhancing existing knowledge to boost efficiency. Finally, exploration leads to disruptive innovations, whereas exploitation results in incremental improvements (62).

**Figure 1:**
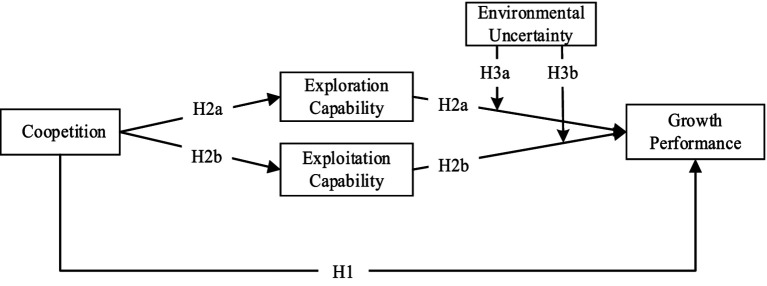
Research model.

In conclusion, drawing on coopetition and dynamic capability theories, this paper contends that coopetition facilitates access to knowledge and resources within the value network and fosters the development of exploration and exploitation capabilities. This enables digital healthcare ventures to navigate complex environments and achieve more rapid growth. In the volatile climate of fast-paced product and service development, environmental uncertainty emerges as a critical external factor influencing the impact of exploration and exploitation capabilities on venture growth. Figure 1 illustrates the theoretical research model that this paper proposes.

### 2.3 Coopetition and growth performance in digital healthcare ventures

While existing research emphasises the coopetition strategies of traditional firms to achieve competitive advantage by leverarging insights from the coopetition theory (12, 13), some scholars advocate for a deeper understanding of how relationships evolve from competition to coopetition among firms in the digital era in order to sustain business growth (24, 25). In today’s environment, digital healthcare ventures attempting to navigate these complexities alone often lack the necessary resources, technology, or knowledge to independently manage service operations (57, 63). As a result, coopetition has become a crucial trend in the strategic development of these ventures, with the resources and knowledge it facilitates playing a significant role in their growth objectives. The conventional healthcare setting, characterised by a scarcity of quality resources, challenging doctor-patient interactions, system inefficiencies, and information asymmetry, limited the main entities to traditional offline interactions (7). Digital technologies have transformed these traditional business models, altering how ventures manage uncertainties in an ever-evolving environment and paving the way to Internet-driven business models like electronic data interchange (EDI) and video teleconferencing (56). Connectivity has thus become a cornerstone of effective strategy implementation for digital healthcare ventures (64). Digital channels facilitate rapid and efficient information dissemination, increasing connectivity among actors in the value network and reducing communication costs (7). This enhanced connectivity allows digital healthcare ventures to transcend traditional constraints of time, space, and geography, sharpening the focus on healthcare processes and optimizing the allocation of healthcare resources (50, 65).

Several studies have explored the effects of coopetition and sought ways for firms to improve their competitive edge through inter-organisational interaction (13). Existing research on coopetition strategy and firm growth in the digital age is still in the early stages, focusing on the relationship between coopetition and firm performance among traditional firms (12, 31, 44). Given their typically small scale, limited resources, and legitimacy, digital healthcare ventures face significant challenges in the competitive healthcare service industry of the digital era (53). By adopting the coopetition strategy, actors in the value network collaborate to explore the market, participate in market competition, improve market competitiveness, and promote enterprise development (26, 66). On the one hand, digital healthcare ventures engage in horizontal coopetition to achieve growth. Horizontal coopetition refers to the cooperation between two companies at the same stage of the value chain to jointly create, produce, and market a new product while competing against other market players (15). In horizontal coopetition, competitors share similar resources, capabilities, and technologies at the same stage of the value chain. They combine these resources to create new resources, resulting in highly collaborative learning among competitors (67). Collaboration among experts in the same field significantly increased opportunities for mutual exchange through knowledge sharing. Competitors pool similar resources and knowledge, which plays a significant role in facilitating venture growth (21, 68). Wu and He (69) argued that tourism companies are compelled to adopt new strategies as horizontal coopetition to foster effective innovation by sharing resources that are otherwise unavailable in the market, enabling individual tourism enterprises to create value and maximise competitiveness. On the other hand, vertical coopetition appears relevant for firm growth. Vertical coopetition is a relationship in which two companies cooperate in complementary ways at different stages of the value chain while competing horizontally in the end market with their respective products (15). Because the ventures lack certain knowledge or proficiency in certain technologies, they utilise their competitors’ expertise and resources to deliver the best end product or service to their customers (4, 43). According to Lechner et al. (29), vertical coopetition serves as a social capital source for a firm’s development, enabling access to more relevant resources and leveraging resource complementarities to boost sales growth.

Therefore, for digital healthcare ventures grappling with the challenges of being new and small, stakeholder cooperation is not just advantageous but essential (50, 70). In the digital age, strategic success hinges less on comparative advantage and more on seizing growth opportunities (71). Ventures that embrace coopetition can accelerate knowledge exchange within value networks, facilitate learning, and share information and resources, thereby reducing healthcare waste, enhancing service quality and efficiency, and effectively cultivating growth opportunities (44).

*H1:* Coopetition has a positive influence on growth performance in digital healthcare ventures.

### 2.4 The mediating effect of exploration and exploitation capabilities

Digital empowerment has shifted the healthcare paradigm from a “hospital-centered” model, which focuses on disease diagnosis and treatment, to one that necessitates digital healthcare ventures to dynamically respond to market trends, embrace a “patient-centered” philosophy, and flexibly tailor their service strategies to user needs and preferences (56). Dynamic capability theory suggests that the growth of digital healthcare ventures relies on two key factors: optimising existing organisational capabilities for efficiency and diverging from established processes and structures to discover new opportunities. This transformative process revolves around the exploration and exploitation capabilities of the ventures (62, 72). Previous studies have found that coopetitive relationships within value networks facilitate the transfer and dissemination of resources and knowledge (12, 73). Additionally, it has been recognised that digital healthcare ventures must possess both exploration and exploitation capabilities to effectively manage their financial performance in the short term while also ensuring long-term growth (74). However, some studies argue that exploration and exploitation serve as key starting points for firm growth while emphasizing the importance of coopetition strategy as a catalyst for this process (75, 76). In the digital era, where the external environment of organisations is marked by unpredictability (24), digital healthcare ventures often lack the market experience of larger companies (3). Using various digital technologies, these ventures operate in an open environment that encourages the search for and acquisition of resources and knowledge. This openness facilitates the emergence of new ideas and interdisciplinary approaches, attracting complementary resources and skills from different groups, and gaining competitive advantages (14, 19). We hypothesize that these capabilities mediate the relationship between coopetition and growth performance in digital healthcare ventures.

Firstly, digital healthcare ventures that embrace coopetition leverage digital technology to exchange information and resources with value network members, enhancing their ability to acquire knowledge and improve exploration capability. This process enables them to master new knowledge and resources through continuous search, discovery, and experimentation. Consequently, they can develop new technologies and products, enter new markets, and capitalise on new opportunities, thereby boosting growth performance (77). Specifically, these ventures build coopetitive relationships with stakeholders, forming digital-based value networks that facilitate dynamic interactions among members. This network deepens their understanding and mastery of external knowledge, breaks conventional thinking, ignites creative sparks, enhances internal innovation drive, and internalises knowledge through feedback and updates. As a result, they absorb new insights, alter cognitive paths, and achieve organisational innovation or value creation, thus enhancing exploration capability (74). Exploration capability empowers digital healthcare ventures to harness digital technologies to access new knowledge and resources, particularly quantifiable and real-time healthcare data and user-generated content. Ventures explore data resources’ potential through analytics, utilise big data to anticipate market trends and user needs, uncover new opportunities, and offer personalised, customised, and experiential digital healthcare services, culminating in exceptional service experiences (4). Ventures proficient in exploration thrive in the complex, dynamic, diverse, and uncertain digital healthcare landscape, ultimately improving growth performance (63, 78). Ferreira et al. (73) found that strategic alliances, as one of the main realisations of coopetition strategies, provide new ventures with access to additional resources and knowledge, enabling them to pursue business opportunities that would otherwise be unreachable. This access allows firms to develop new products, services, and technologies. Seepana et al. (75) identified the need to develop managerial ambidexterity to address increasing supply chain complexity (79), enabling managers to explore solutions while utilising existing knowledge to benefit their companies. As a result, the coopetition strategy is gaining attention as a crucial factor in improving growth performance. Consequently, digital healthcare ventures are likely to strengthen their competitive advantages by exploration capability with various partners to create superior value.

Secondly, digital healthcare ventures cannot solely rely on exploration capabilities for growth; they must also improve their exploitation capabilities. This process involves extending existing knowledge, technologies, and paradigms through iterative refinement and application to foster growth (80). Specifically, collaboration with value network members and the boundary-spanning nature of digital technology promote the flow of resources, enabling ventures to assimilate mature knowledge from coopetition. This expertise is then applied as an integral resource for growth, maintaining technological prowess to continuously reorganise, integrate, and deploy external knowledge to refine their healthcare products, services, and experiences, thus bolstering exploitation capability (74). Exploitation capability allows digital healthcare ventures to use existing knowledge to polish competencies along their original development trajectory, deepening the utilisation of existing resources and capabilities (74). They particularly exploit the value of expansive, multi-source, heterogeneous, and high-dimensional patient behavioral data to improve application capabilities. This data is invaluable for patient relationship management, and ventures quickly adapt their offerings based on user feedback to meet the functional needs of convenience, affordability, and efficiency, thereby increasing patient satisfaction and loyalty (78). Seepana et al. (75) highlighted coopetition is particularly associated with managerial ambidexterity, translating combinations of resources and capabilities into potential performance benefits. Yang and Zhang (26) emphasised coopetition provides many learning opportunities for ventures as it promotes knowledge sharing among firms and creates a common knowledge base. By engaging in coopetition activities, ventures can utilise existing knowledge, which may become a source of competitive advantage and support the growth of ventures. Through the above discussion, it is expected that ventures will seek exploitation capabilities based on coopetition strategies to promote growth performance.

In summary, this paper posits that exploration and exploitation capabilities are the vital conduits linking coopetition to the growth performance of digital healthcare ventures. Thus, we propose the following hypotheses:

*H2a:* Exploration capability plays a mediating role in the relationship between coopetition and growth performance in digital healthcare ventures.

*H2b:* Exploitation capability plays a mediating role in the relationship between coopetition and growth performance in digital healthcare ventures.

### 2.5 The moderating effect of environmental uncertainty

Digitisation leads to change and innovation in the business environment, offering substantial potential to improve company efficiency and foster business innovation (24). In the swiftly evolving landscape of digital technology, the healthcare service industry is undergoing a gradual yet profound transformation, confronting the inevitability of a digital overhaul. Innovations like the Internet of Medical Things (IoMT) and healthcare wearable devices (HWDs) have become indispensable factors in digital health management, significantly impacting the industry’s trajectory (81). Digital healthcare ventures, often characterised by their modest scale and limited risk tolerance, find themselves particularly vulnerable to the volatile external environment. Thus, in a business landscape where digital technologies intricately intertwine with healthcare services, the cultivation of exploration and exploitation capabilities becomes vital for their survival and success (82). In this context, environmental uncertainty refers to the rapidity and intensity of technological and market changes within an industry (36, 58, 83). While existing research has extensively explored the impact of environmental uncertainty on firm performance and innovative development in traditional contexts, there remain disagreements in the literature (33–35, 37). This has resulted in a lack of exploration of the mechanisms of environmental uncertainty enabling firm growth in digital contexts (36). This paper, therefore, views environmental uncertainty as a significant contingency variable affecting the growth performance of digital healthcare ventures.

In an environment characterised by continuous innovation in products, services, and experiences, digital healthcare ventures face the challenge of identifying new business opportunities through emerging technologies and knowledge (62). Coreynen et al. (58) suggested that exploration capability is a dynamic capacity that empowers organisations to recognise new market possibilities and foster growth by pioneering product, service, and technology ideas. This ability positions ventures for growth, particularly in uncertain climates. High environmental uncertainty risks rendering current offerings obsolete and stifling innovation if ventures adhere to the status quo (78). To stay ahead, digital healthcare ventures must eschew inertia, escape the “success trap,” and actively harness technologies like healthcare big data to tap into the value of new knowledge and resources, thereby developing fresh markets and innovative digital healthcare solutions that prioritize patient comfort and satisfaction (63). Wang et al. (35) confirmed that due to the high level of process integration within IT-enabled firms, high levels of automation may hinder rather than enhance exploitative innovation in uncertain environments. Conversely, Coreynen et al. (58) argued that exploitation capability enables ventures to capitalise on existing knowledge to enhance current solutions, increase efficiency, and drive growth. In environments with lower uncertainty, ventures face higher risks and costs associated with an innovation model rooted in exploitation capability, which can impede the sustainability of high R&D investment in new products and technologies (63). In such scenarios, ventures may benefit from refining existing digital healthcare products, services, and technologies, as well as improving the reliability of current solutions to reduce costs (62, 78). Wang et al. (35) postulated that firms tend to pursue more aggressive strategies and innovations in less certain environments. Therefore, exploitation capability tends to be more advantageous than exploration capability in relatively stable environments.

To summarise, the beneficial impact of exploration capability on the growth performance of digital healthcare ventures is amplified in highly uncertain environments, whereas the positive influence of exploitation capability on growth performance diminishes. Accordingly, we propose the following hypotheses:

*H3a:* Environmental uncertainty has a positive effect on the relationship between exploration capability and growth performance in digital healthcare ventures.

*H3b:* Environmental uncertainty has a negative effect on the relationship between exploitation capability and growth performance in digital healthcare ventures.

## 3 Methodology

### 3.1 Data collection and the sample

In this study, a questionnaire survey was utilised to collect relevant data. To test the hypotheses, digital healthcare ventures in China were selected as the sample source, where inter-organisational cooperation and competition are interwoven for economic and social growth (84). As a dynamic emerging market with rapid economic growth and vast market potential (24), China has seen significant government efforts aimed at promoting the transformation and upgrading of the healthcare industry. Consequently, the digital healthcare sector has experienced remarkable growth and has emerged as a new driver of China’s digital economy (85). The study utilised the Index of Regional Innovation and Entrepreneurship in China (IRIEC), a publication by Peking University’s Center for Enterprise Research, to differentiate regions based on their levels of innovation and entrepreneurial activity. It categorised areas with high levels of such activities, like Guangdong, Jiangsu, and Zhejiang provinces, as well as those with lower levels, such as Hainan and Heilongjiang provinces. We employed a random sampling approach, selecting incubators and accelerators in these areas based on our strong early-stage connections with local government bodies, industry associations, and other entities. To increase sample representativeness, the study included ventures from various regions, extending the sample’s scope (86). We used GPower software to estimate the required sample size for this study. This study used linear regression analysis to analyse the effects of coopetiton, exploration and exploitation capabilities, and environmental uncertainty on growth performance. Therefore, we chose the F-test (linear multiple regression) (87). We set the power at 95% and the alpha value at 0.05. Based on these parameters, a minimum sample of 129 subjects is required. We collected a total of 338 effective questionnaires for this study, fulfilling the required minimum number of respondents as calculated by GPower.

Adopting a hybrid method of internet and traditional paper-based questionnaires, this study utilised both online and on-site paper questionnaires from March to August 2023, with online methods predominating and offline questionnaires serving as supplements. Prior to data collection, researchers received training on survey techniques and protocols. Potential respondents were contacted through phone or WeChat to explain the study’s purpose and methods and to schedule their participation, enhancing their willingness to participate and potentially increasing response rates. Respondents were assured of their privacy and informed that the data would be used solely for research purposes, with no commercial intent. They were told that personal information would remain confidential and that the results would be shared upon request. During the questionnaire administration, researchers explained the process, documented notes, and addressed any inquiries. If respondents could not complete the questionnaire on-site, an electronic version was provided, with a request to return it via email within a set timeframe. To ensure the reliability and validity of the measurements, the questionnaire was prepared by modifying it appropriately for this study in digital healthcare contexts based on the items used in the previous study. It was first written in English and then translated into Chinese with the help of a professional bilingual translator. The back translation method was employed to refine the scales until the Chinese and English versions were closely aligned. The questionnaire was reviewed by three field experts for its structure, readability, and completeness, resulting in minor adjustments based on their suggestions. After developing the draft questionnaire, we conducted a pilot test with 50 questionnaires to verify the appropriateness of each question, resulting in the final questionnaire. We distributed the finalised questionnaires to at least two firm managers per firm. Of the 735 questionnaires distributed to 300 ventures, 514 questionnaires were returned, resulting in a 69.93% response rate. After eliminating all invalid cases such as incomplete answers (e.g., a significant portion of the questionnaire remains unanswered), straight-lining (e.g., a respondent provides identical answers), non-target populations (e.g., individuals who do not belong to digital healthcare ventures), and internal inconsistencies (e.g., apparent inconsistencies or logical errors within the questionnaire responses), 338 effective questionnaires remained, resulting in an effective response rate of 45.99%. Given that the typical response rate of web-based surveys ranges from 10 to 15% (88), the achieved response rate strongly supports the adequacy of the targeted participants and the relevance of our study. The majority of responding companies were founded by males, with an average company age of 3.97 years, and 60.1% of the enterprises were established for less than 4 years. Most responding companies (80.5%) were small-sized enterprises with fewer than 100 employees, predominately private, followed by foreign-owned enterprises, state-owned enterprises, and joint enterprises. Geographically, 22.2% of the enterprises were located in Guangdong Province, 15.4% in Jiangsu Province, 14.5% in Zhejiang Province, 25.1% in Hainan Province, and 22.8% in Heilongjiang Province. Also, an independent sample t-test that compared the means of early and late responses on the variable “firm size” did not find any statistically significant differences. This suggests that non-response bias is not a problem in this study (89).

### 3.2 Constructs and measurement

All constructs were assessed using a five-point Likert scale, ranging from “Strongly Disagree” to “Strongly Agree,” with corresponding values of 1 to 5 points.

Independent variable-coopetition: This construct reflects the need for digital healthcare ventures to collaborate with various stakeholders, including similar enterprises, hospitals, drug distributors, financial institutions, insurance companies, governments, and users, in order to create value networks in the digital economy (90). We employed the scale by Bouncken and Fredrich (14), which demonstrated satisfactory reliability with a Cronbach’s coefficient of 0.769.

Dependent variable-growth performance: recognising the limited applicability of traditional financial indicators to digital healthcare ventures, which often exhibit shorter lifespans and substantial initial investments, this construct focuses on metrics such as sales and profit growth rates as more indicative of success (65, 78). The scale by Wang et al. (91) was used, yielding a Cronbach’s α of 0.789, indicating good reliability.

Mediator variable-exploration and exploitation capability: Exploration and exploitation capabilities are critical in the entrepreneurial process of digital healthcare ventures (92). The study adopted the scale by Cheng and Sheu (74), with Cronbach’s α coefficients of 0.828 and 0.858, respectively, reflecting the scale’s reliability.

Moderator variable-environmental uncertainty: Extensive research suggests that a company’s external environment is an important factor that can affect venture growth (81). Environmental uncertainty was measured using scales from Lissillour et al. (37), resulting in a Cronbach’s α of 0.778, confirming the scale’s reliability.

Controls: Given their documented influence on venture growth performance (14, 43, 66), we included founder gender, firm age, firm size, and firm ownership as control variables. Firm age was determined by years since establishment, and firm size by employee count (66). Firm ownership was defined by the dominant shareholder type, including private, joint, state-owned, and foreign-owned enterprises, with a dummy variable for firm ownership (84).

## 4 Result

### 4.1 Reliability and validity of measures

SPSS 26.0 and AMOS 26.0 were utilised to assess the reliability and validity of the constructs in this study. Consistent with Anderson and Gerbing (93), we first conducted a measurement model test before evaluating the conceptual model. Exploratory factor analysis (EFA) using SPSS 26.0 identified the latent constructs, employing principal axis factoring and varimax rotation with Kaiser Normalisation. Factors with eigenvalues greater than one were retained, while those with eigenvalues less than one were considered insignificant. The EFA extracted five factors with eigenvalues exceeding 1.0, explaining 68.029% of the variance in the data: coopetition (18.425%), exploration capability (16.802%), exploitation capability (11.470%), growth performance (11.181%), and environmental uncertainty (10.151%). The Kaiser-Mayer-Olkin (KMO) test yielded a value of 0.885, above the threshold of 0.8, and Bartlett’s test of sphericity achieved a chi-square of 3011.506 with 171 degrees of freedom, *p* < 0.01, indicating suitability for factor analysis (94). Table 1 presents a prerequisite analysis for descriptive statistics and confirmatory factor analysis. The mean of all observed variables ranged from 3.590 to 4.371, with a standard deviation spanning from 0.596 to 0.803. All standard factor loadings were 0.6 or higher, exceeding the threshold required for confirmatory factor analysis (*p* < 0.001) (93). Table 2 shows the measurement model’s fit for the five constructs using AMOS 26.0 software. The measurement model demonstrates a good fit to the data: χ^2^(142) = 345.881, *p* < 0.001, χ^2^/df = 2.436, CFI = 0.930, TLI = 0.916, IFI = 0.931, RMSEA = 0.065, and SRMR = 0.060. Comparative analyses with alternative models (four-factor, three-factor, two-factor, and one-factor) supported the superiority of the five-factor model. Table 3 evaluates the correlation, convergence, and discriminant validity among factors. All Cronbach’s alpha values exceeded the 0.7 threshold for internal consistency reliability, confirming the acceptability of the measures (95). Composite reliability (CR) values surpassed the 0.70 benchmark, and average variance extracted (AVE) values exceeded 0.50, indicating that convergent validity was achieved (95). The AVE square root values were higher than the inter-construct correlation coefficients, which showed that it was a good discriminant (96).

**Table 1:** Descriptive statistics and confirmatory factor loadings.

Constructs (source)	Item	SFL	Mean	SD
Coopetition (14)	We are in close competition with our partners.	0.749	4.400	0.708
	We collaborate with competitors to achieve a common goal.	0.701	4.350	0.700
	An active competition with our collaborators is important to us.	0.728	4.360	0.751
Exploration capability (74)	Acquired digital healthcare technologies and skills entirely new to the firm.	0.889	3.480	1.025
	Learned digital healthcare product development processes-related knowledge entirely new to the industry.	0.654	3.930	0.855
	Acquired completely new skills and organisational management in the field of digital healthcare (market trends, project management,…).	0.686	3.520	0.966
	Learned new knowledge in areas such as funding new technologies, staffing R&D function, and training and development of healthcare knowledge and skills for the first time.	0.798	3.670	0.821
	Strengthened innovation knowledge in the field of digital healthcare where it had no prior experience.	0.619	3.570	0.720
Exploitation capability (74)	Upgraded current digital healthcare knowledge and skills for familiar products, service and technologies.	0.841	3.810	1.075
	Invested in enhancing knowledge in exploiting mature technologies that improve the efficiency of its current operations and healthcare services.	0.788	3.570	0.997
	Enhanced competencies in searching for solutions to customer problems that are near to existing solutions rather than completely new solutions.	0.651	3.730	0.860
	Upgraded knowledge in digital healthcare product and service design and development processes in which the firm already possesses significant experience.	0.660	3.710	1.056
	Strengthened our knowledge and skills for projects that improve efficiency of existing healthcare activities.	0.798	3.670	0.825
Growth performance (91)	Sales growth speed.	0.639	3.570	0.922
	Profit growth speed.	0.909	3.550	1.061
	Market share growth speed.	0.660	3.640	0.881
Environmental uncertainty (37)	The speed of technological change in the industry is very fast.	0.763	4.270	0.817
	The products and services in the industry are updated very quickly.	0.645	3.660	0.858
	Patient needs are changing rapidly and becoming increasingly diverse and individualized.	0.814	4.230	0.807

SFL = standardized factor loading, all SFLs are significant (*p* < 0.000). SD standard deviation.

**Table 2:** Confirmatory factor analysis.

Model	χ^2^(*df*)	χ^2^/*df*	IFI	TLI	CFI	RMSEA	SRMR
5-factor model (X, M_1_, M_2_, Y, W)	345.881 (142)	2.436	0.931	0.916	0.930	0.065	0.060
4-factor model (X, M_1_, M_2_ + Y, W)	511.586 (146)	3.504	0.875	0.853	0.874	0.086	0.072
3-factor model (X + M_1_, M_2_ + Y, W)	730.835 (149)	4.905	0.801	0.770	0.800	0.108	0.090
2-factor model (X + M_1_, M_2_ + Y + W)	977.629 (151)	6.474	0.717	0.678	0.716	0.127	0.106
1-factor model (X + M_1_ + M_2_ + Y + W)	1203.244 (152)	7.916	0.641	0.593	0.638	0.143	0.106

X: Coopetition; Y: Growth performance; M_1_: Exploration capability; M_2_: Exploitation capability; W: Environmental uncertainty.

**Table 3:** Descriptive statistics and confirmatory factor loadings.

	1	2	3	4	5
Coopetition	1				
Exploration capability	0.375**	1			
Exploitation capability	0.360**	0.529**	1		
Growth performance	0.313**	0.560**	0.557**	1	
Environmental uncertainty	0.359**	0.318**	0.312**	0.232**	1
Cronbach’s α	0.769	0.828	0.858	0.789	0.778
Composite reliability	0.770	0.853	0.865	0.786	0.787
AVE	0.528	0.542	0.565	0.557	0.554
SQRT (AVE)	0.727	0.736	0.752	0.746	0.744

AVE = average variance extracted, SQRT = square rooted.

### 4.2 Common method bias

Common method bias (CMB) arises when variables are measured contemporaneously using a single instrument (97). We implemented both procedural and statistical controls to mitigate CMB. Procedurally, we assured respondents of anonymity, clearly communicated the purpose of the questionnaire, and collected data from at least two firm managers per firm (84). We conducted Harman’s single factor test statistically to assess potential CMB. The first factor extracted from exploratory factor analysis accounted for less than the critical threshold of 50% (98). Additionally, as indicated in Table 2, Harman’s single-factor model was tested and found to fit the data poorly: χ^2^(152) = 1203.244, *p* < 0.001, χ^2^/df = 7.916, RMSEA = 0.143, SRMR = 0.106, CFI = 0.638, IFI = 0.641, TLI = 0.593. The single-factor model fit was inferior to our measurement model, suggesting that CMB was not a significant concern in this study (99).

### 4.3 Descriptive statistical analysis and correlations

Table 3 presents the descriptive statistics and correlations, offering support for the variables and hypotheses’ credibility. These correlations indicate significant relationships among coopetition, exploration capability, exploitation capability, environmental uncertainty, and growth performance. These findings align with the research direction and provide a solid basis for hypothesis testing. Given that all correlation coefficients between latent variables were significantly positive (*p* < 0.05), nomological validity was verified (95).

### 4.4 Results of analysis

Before running the model in SPSS 26.0, we assessed the threat of collinearity using the summated scores of the latent variables. The highest variance inflation factor (VIF) for the exogenous variables was 1.495, well below the threshold of 5, indicating no significant collinearity concerns in this study (95, 100). Regression analysis and bootstrap methods were applied to test the research hypotheses. The results showed that coopetition had a significant and positive effect on growth performance (β = 0.310, *p* < 0.001). This supports the assertion that coopetition has a positive effect on growth performance in digital healthcare ventures, which proves H1.

Using Hayes’ PROCESS plug-in, we conducted bootstrap analysis with 5,000 iterations for mediation and moderated mediation path tests in Table 4. We employed Model 4 to assess the mediating effects of exploration and exploitation capability, while controlling for variables such as founder gender, firm age, firm size, and firm ownership. Exploration capability positively mediates coopetition’s effect on growth performance (β = 0.177, *p* < 0.05), as the 95% confidence interval {0.093, 0.262} did not include 0. Similarly, exploitation capability positively mediates coopetition’s effect on growth performance (β = 0.169, *p* < 0.05), as the 95% confidence interval {0.087,0.244} did not include 0. The direct effect of coopetition on growth performance was 0.072, and the confidence interval is {−0.051,0.195} including 0, indicating full mediation. Thus, H2a and H2b are supported.

**Table 4:** Mediation and moderated mediation analyzes.

Effect	Mediation model			Moderated mediation model		
	Estimate	SE	[LLCI,ULCI]	Estimate	SE	[LLCI,ULCI]
X➔M_1_	0.422	0.058	[0.308,0.536]	0.422	0.058	[0.308,0.536]
X➔M_2_	0.459	0.067	[0.328,0.590]	0.459	0.067	[0.328,0.590]
M_1_➔Y	0.420	0.061	[0.301,0.539]	0.426	0.064	[0.301,0.551]
M_2_➔Y	0.367	0.053	[0.264,0.471]	0.388	0.053	[0.283,0.492]
X➔Y	0.072	0.063	[−0.051,0.195]	0.076	0.074	[−0.070,0.221]
X➔ M_1_ ➔ Y	0.177	0.044	[0.093,0.262]	0.094	0.042	[0.011,0.180]
X➔ M_2_ ➔ Y	0.169	0.040	[0.087,0.244]	−0.103	0.047	[−0.195,−0.008]
W➔Y				-0.008	0.054	[−0.115,0.099]
M_1_ × W➔Y				0.223	0.072	[0.083,0.364]
M_2_ × W➔Y				−0.226	0.082	[−0.387,-0.064]
Controls						
Founder gender➔Y	0.001	0.071	[−0.138,0.140]	0.003	0.071	[−0.136,0.142]
Firm size➔Y	0.029	0.029	[−0.029,0.086]	0.025	0.029	[−0.108,0.089]
Firm age➔Y	−0.023	0.014	[−0.050,0.004]	−0.022	0.014	[−0.049,0.005]
Firm ownership➔Y	−0.005	0.050	[−0.104,0.095]	−0.010	0.050	[−0.033,0.082]

X: Coopetition; Y: Growth performance; M_1_: Exploration capability; M_2_: Exploitation capability; W: Environmental uncertainty.

For moderating effects, Model 14 in the PROCESS plug-in was utilised, considering the same control variables. The interaction of exploration capability and environmental uncertainty had a significant positive effect on growth performance (β = 0.223, *p* < 0.05), as the 95% confidence interval {0.083, 0.364} did not include 0, affirming H3a. Conversely, the interaction between exploitation capability and environmental uncertainty had a significant negative effect on growth performance (β = −0.226, *p* < 0.05), with a confidence interval of {−0.387, −0.064} excluding 0, thereby supporting H3b.

### 4.5 Robustness checks

We examined the robustness of our findings for equivalent models. Firstly, we assessed Model 59, a full moderated mediation model, by including moderations of the first and second stages, and direct effects. Table 5 displays the results. In the direct effect, we found that environmental uncertainty did not have a moderating effect on the relationship between coopetition and growth performance (β = −0.037, *p* > 0.1). In the first stage, environmental uncertainty negatively moderates the effects of coopetition on exploration capability (β = −0.279, *p* < 0.05) and exploitation capability (β = −0.202, *p* < 0.05). Moreover, we found that environmental uncertainty positively moderated the relationship between exploration capability and growth performance (β = 0.234, *p* < 0.05) and negatively moderated the relationship between exploitation capability and growth performance (β = −0.201, *p* < 0.05), which is consistent with our findings. Moreover, we examined whether the positive direct effects of the second stages of exploitation capability-exploration capability are moderated by each other. In Table 6, the results show that there is no significant interaction effect between exploration capability and exploitation capability on growth performance (β = 0.027, *p* > 0.1). Furthermore, we explored a 3-way interaction of exploitation capability, exploration capability, and environmental uncertainty. In Table 7, the results indicated no significant interaction effect between exploration capability, exploitation capability, and environmental uncertainty on growth performance (β = 0.098, *p* > 0.1). These results are generally consistent with our findings, demonstrating the robustness and validity of the findings in this study.

**Table 5:** The full moderated mediation model.

Effect	Moderated mediation model		
	Estimate	SE	[LLCI,ULCI]
X➔M_1_	0.116	0.067	[−0.016,0.204]
X➔M_2_	0.201	0.080	[0.044,0.358]
M_1_➔Y	0.425	0.064	[0.300,0.550]
M_2_➔Y	0.384	0.054	[0.279,0.490]
X➔Y	0.076	0.074	[−0.070,0.222]
X➔ M_1_ ➔ Y	0.049	0.028	[−0.001,0.107]
X➔ M_2_ ➔ Y	0.077	0.034	[0.013,0.148]
W➔Y	−0.018	0.059	[−0.133,0.098]
X × W➔Y	−0.037	0.092	[−0.219,0.144]
X × W➔M_1_	−0.279	0.043	[−0.364,-0.194]
X × W➔M_2_	−0.202	0.052	[−0.304,-0.101]
M_1_ × W➔Y	0.234	0.076	[0.084,0.384]
M_2_ × W➔Y	−0.201	0.101	[−0.401,-0.020]
Controls			
Founder gender➔Y	0.001	0.071	[−0.140,0.140]
Firm size➔Y	0.024	0.029	[−0.033,0.082]
Firm age➔Y	−0.022	0.014	[−0.049,0.005]
Firm ownership➔Y	−0.011	0.050	[−0.110,0.088]

X: Coopetition; Y: Growth performance; M_1_: Exploration capability; M_2_: Exploitation capability; W: Environmental uncertainty.

**Table 6:** The 2-way interaction of exploitation capability and exploration capability.

Effect	Moderated mediation model		
	Estimate	SE	[LLCI,ULCI]
X➔M	0.339	0.061	[0.219,0.459]
M➔Y	0.427	0.062	[0.305,0.550]
X➔Y	0.093	0.074	[−0.052,0.237]
X➔ M ➔ Y	0.145	0.037	[0.074,0.222]
W➔Y	0.370	0.053	[0.265,0.475]
M × W➔Y	0.027	0.064	[−0.099,0.152]
Controls			
Founder gender➔Y	0.001	0.071	[−0.139,0.141]
Firm size➔Y	0.029	0.030	[−0.029,0.087]
Firm age➔Y	−0.023	0.014	[−0.050,0.004]
Firm ownership➔Y	−0.007	0.051	[−0.107,0.093]
Environmental uncertainty➔Y	−0.008	0.060	[−0.126,0.109]

X: Coopetition; Y: Growth performance; M: Exploration capability; W: Exploitation capability.

**Table 7:** The 3-way interaction of exploitation capability, exploration capability and environmental uncertainty.

Effect	Moderated mediation model		
	Estimate	SE	[LLCI,ULCI]
X➔M	0.422	0.058	[0.308,0.536]
M➔Y	0.419	0.064	[0.293,0.544]
X➔Y	0.074	0.075	[−0.074,0.222]
X➔ M ➔ Y	0.177	0.044	[0.091,0.264]
W_1_➔Y	0.377	0.054	[0.272,0.482]
W_2_➔Y	−0.056	0.077	[−0.208,0.096]
M × W_1_ × W_2_➔Y	0.098	0.067	[−0.033,0.230]
Controls			
Founder gender➔Y	0.012	0.071	[−0.128,0.152]
Firm size➔Y	0.021	0.029	[−0.037,0.078]
Firm age➔Y	−0.022	0.014	[−0.049,0.005]
Firm ownership➔Y	−0.017	0.050	[−0.116,0.082]

X: Coopetition; Y: Growth performance; M: Exploration capability; W_1_: Exploitation capability; W_2_: Environmental uncertainty.

## 5 Discussion

### 5.1 Discussion of findings

Amidst the ongoing wave of digitalisation, the proliferation of information technologies such as big data, cloud computing, and artificial intelligence has accelerated the digitisation of healthcare ventures, positioning digital healthcare as a pivotal driver for the rapid development of the digital economy (65, 78). To elucidate the relationship between coopetition and growth performance in digital healthcare ventures, this paper delves into the underlying mechanisms and boundary conditions informed by coopetition theory and dynamic capabilities theory. Specifically, the study reveals: (1) Coopetition directly and positively impacts growth performance in digital healthcare ventures; (2) Coopetition indirectly influences growth performance through exploration and exploitation capabilities in digital healthcare ventures; and (3) Environmental uncertainty strengthens the positive effect of exploration capability on growth performance while weakening the impact of exploitation capability in digital healthcare ventures.

Based on coopetition theory, H1 posits that coopetition benefits growth performance in digital healthcare ventures. It suggests that for sustained growth, such ventures should foster coopetitive relationships with multiple stakeholders within the value network (83). Ritala (18) argued that competitors often share a similar common logic and have sufficiently similar resources, leading to increased relative absorptive capacity and value creation in certain contexts. This, in turn, provides them with the ability and motivation to integrate resources, further contributing to firms’ innovative output and market performance in general. Lee and Roh (24) indicated that firms in emerging markets strengthen their competitive advantages by sharing resources and capabilities with various partners and competing to create superior value. Coopetition strategies provide multiple opportunities to exchange knowledge with non-competitive and competitive partners, and they are critical to adding value and achieving growth performance (25). However, Westra et al. (101) found that different organisations share different specialists with competitors and non-competitors, and healthcare organisations in developed economies are reluctant to share their most specialised human resources, seemingly protecting their competitive advantage, which limits the full knowledge-sharing potential of this type of inter-organisational relationship. Therefore, in future research, whether coopetition in healthcare benefits patients remains a common concern for both developing and developed economies. Our study argues that digital healthcare ventures leverage these value networks as platforms for stakeholder collaboration, echoing previous research that aligns coopetition with enhanced firm growth and performance (12, 24, 29, 68). While some scholars have debated the impact of different intensities and phases of coopetition on performance and innovation (17, 31), Bendig et al. (12) investigated the effect of coopetition intensity in different phases of new product development alliances on focal firms’ innovation outcomes. The results show that the early and later phases of coopetitive new product development pose different benefits and risks for different types of innovation. Bouncken et al. (102) also examined, more specifically, the different value capture processes adopted by coopetition for mature SMEs and small SMEs. This paper views coopetition for digital healthcare ventures holistically, acknowledging its overall positive effect.

Regarding H2, this paper discusses how exploration and exploitation capabilities mediate the relationship between coopetition and growth performance in digital healthcare ventures, in line with dynamic capabilities theory. Lee and Roh (24) aim to review sustainable growth methods through strategic behavior based on a dynamic capability perspective. Similarly, this study suggests that coopetition can indirectly enhance growth performance through these dynamic capabilities, which involve learning about knowledge dynamics within and outside the organisation (103). This resonates with findings from Xia and Roper (104), Lisboa et al. (105), and Cheng and Sheu (74), who have identified these capabilities as pivotal in enhancing firm performance and growth. From a resource-based point of view, coopetition networks are strategic resources that come from unique historical experiences and knowledge. They help the development of dynamic capabilities, which in turn supports growth. Additionally, this study investigates whether coopetition contributes to developing exploration and exploitation capabilities. Our results reveal that digital healthcare ventures armed with coopetition strategies can be ambidextrous organisations. This implies that digital healthcare ventures can effectively improve their exploration and exploitation capabilities through key coopetition strategies such as strategic alliances, joint venture agreements, and equity participation. As argued by Monticelli et al. (106), competing firms collaborate to overcome challenges by exploring and exploiting opportunities, taking risks, and developing solutions in highly regulated industries, such as healthcare in developing economies. Our findings are consistent with previous studies by Ferreira et al. (73) and Strese et al. (76), asserting that firms should participate in a coopetition strategy to promote organisational ambidexterity. Furthermore, we find that exploration and exploitation capabilities have a significant positive impact on growth performance in digital healthcare ventures. According to the dynamic capabilities theory, a firm’s fundamental competencies should produce short-term competitive positions that can transform into a long-term competitive advantage (55). In line with the dynamic capabilities theory, this finding supports previous research showing that firms with exploration and exploitation capabilities have high survival rates, growth, and performance (38, 107, 108).

In the H3 discussion, the study proposes that environmental uncertainty significantly moderates the influence of exploration and exploitation capabilities on the growth performance in digital healthcare ventures. As environmental uncertainty increases, the beneficial impact of exploration capability on growth performance intensifies, while the positive contribution of exploitation capability diminishes. Firms need to adapt to changes in the external environment and continuously adjust their strategies, capabilities, and resources to meet the challenges. Ritala (18) posited that in situations where time and speed are critical elements and the required knowledge quickly becomes outdated, as is the case in the era of digital healthcare, Lee and Roh (24) stated that in a rapidly changing business environment, firms should integrate and reconfigure internal and external resources in response to changes in the environment as they develop their core competencies to gain a competitive advantage. Our study aligns with findings from Yuen et al. (109) and Temouri et al. (34), suggesting that ventures, facing constant market and demand fluctuations, rely on exploration capabilities to embrace disruptive innovation and manage change. Conversely, an excessive reliance on exploitation capabilities, which focus exclusively on internal efficiency in such an uncertain environment, may induce complacency and path dependency. This, in turn, could potentially impede long-term development, especially for digital healthcare ventures in the Healthcare 4.0 era.

In the robustness analysis, we observe that environmental uncertainty negatively moderates the relationship between coopetition and both exploration capability and exploitation capability. Most digital healthcare ventures grapple with the “liability of newness and smallness” (5, 111), facing considerable challenges due to the pressures to enhance efficiency and lower prices amidst high environmental uncertainty. This scenario often results in tighter margins and reduced organisational slack. Cultivating such high-risk and high-cost exploration capabilities through a coopetition strategy would considerably harm the survival of digital healthcare ventures in their early growth stages (107). Therefore, environmental uncertainty negatively moderates the relationship between coopetition and exploration capability. Similarly, environmental uncertainty has a negative effect on the relationship between coopetition and exploitation capability in digital healthcare ventures. In an environment of high uncertainty, digital healthcare ventures seeking to exploitation capability always emphasise the use of existing technology and knowledge, potentially hindering the acquisition of new knowledge (74). This limited accumulation of new knowledge and technology iteration could quickly render existing products, services, and solutions obsolete, preventing ventures from growing in a rapidly changing digital environment. Furthermore, we also find that there is no significant interaction effect between exploration capability and exploitation capability on growth performance. This suggests that the simultaneous pursuit of high levels of exploration and exploitation capabilities, known as the combined dimension of ambidexterity (CD), may not be conducive to the growth of digital healthcare ventures. Given their resource constraints, these ventures face great difficulties in carrying out exploitation and exploration at a high level simultaneously. Instead, adopting the balance dimension of ambidexterity (BD), where firms cultivate exploration and exploitation capabilities, focusing on an even match between the strengths and weaknesses of the two capabilities, seems more appropriate for them. Cao et al. (112) suggested that BD is more beneficial to resource-constrained firms, whereas CD is more beneficial to firms having greater access to internal and/or external resources. Similarly, as Asif (60) argued, successful firms initially balance exploration and exploitation through “punctuated ambidexterity,” alternating between the two so that exploitation follows exploration and vice versa.

### 5.2 Theoretical implications

Theoretically, the evidence from this research has significant implications for existing management theory. Firstly, the findings suggest that coopetition is the antecedent factor of growth performance in digital healthcare ventures. Coopetition improves risk sharing, reduces costs, and increases access to resources and markets, particularly knowledge. It is understood to achieve greater and reciprocal advantages for every firm involved in an inter-organisational relationship (14, 16, 17). Although coopetition is beneficial for firms to achieve sustainable competitive advantage, there remains a dearth of understanding regarding its efficacy in fostering growth performance, especially for digital ventures grappling with challenges such as the liability of newness and smallness (29, 32, 102). As such, this research enriches the literature on growth performance from the theoretical perspective of coopetition. Moreover, it is critical to establish relationships between coopetition and established constructs in other management domains (25, 84). This research, which links coopetition with venture growth performance in the healthcare industry, also contributes to extending coopetition theory in a highly regulated industry.

Secondly, the results show that ambidextrous capability plays a full mediating role in the relationship between coopetition and growth performance in digital healthcare ventures. This research delves into the mechanism by which coopetition impacts growth performance in digital healthcare ventures, pinpointing the full mediating roles of exploration and exploitation capabilities. Additionally, it fosters further convergence between the theories of coopetition and dynamic capabilities. Although prior studies have examined the impact of coopetition on outcomes such as firm performance and innovation through lenses like organisational learning, conflict, and knowledge creation (12, 43, 66), little research has focused on how coopetition impacts growth performance in digital healthcare ventures. To sum up, this study fills in the blanks about the relationship between coopetition and growth performance in digital healthcare ventures by suggesting that exploration and exploitation capabilities act as mediators. This adds to the theoretical discussion on ambidextrous capabilities by creating the theoretical link between coopetition and growth performance in digital healthcare ventures.

Thirdly, we provide unique insights into the external environmental conditions moderating the effectiveness of ambidextrous capability on growth performance in digital healthcare ventures. The study enriches research on the boundaries of ambidextrous capabilities in digital healthcare by elucidating the distinct roles of exploration and exploitation capabilities on growth performance under environmental uncertainty. Although previous research has delved into the moderating effects of external environmental factors, such as environmental dynamics and munificence, and internal strategic decisions, like entrepreneurial orientation and market orientation, on the relationship between ambidextrous capabilities and firm performance (110–114), little is known about how to leverage ambidextrous capability for growth performance in digital healthcare ventures. Our research suggests that growth performance in digital healthcare ventures facing higher environmental uncertainty is more dependent on exploration capabilities than exploitation capabilities. As Lee et al. (115) suggest, exploitation and exploration hold differing values contingent on the environment. Therefore, our findings add value to the growth performance literature by exploring the moderating role of environmental uncertainty in a different way in the digital healthcare sector.

### 5.3 Managerial implications

Our results also provide digital healthcare ventures with practical guidance on how to effectively use coopetition for growth performance in challenging business environments. Firstly, this research suggests that coopetition is a useful strategic decision logic for digital healthcare ventures to increase growth performance, particularly in such business environments characterised by strong resource constraints and high uncertainty. Throughout their growth process, digital healthcare ventures should proactively engage in cooperation with governments, healthcare providers, and patients, all closely interlinked within the value network. By integrating resources, these subjects can generate synergistic effects that exceed the sum of their parts. Therefore, it’s crucial for digital healthcare ventures to understand and embrace the concept of coopetition, cultivate a coopetitive mindset, enhance their coopetition capabilities, set clear coopetition goals based on growth prospects, establish robust relationships within the value network, thus promoting the sustainable development of the healthcare industry, and improve healthcare service quality.

Secondly, a valuable insight from this research is that coopetition helps digital healthcare ventures engage in ambidextrous capabilities effectively. Coopetition allows digital healthcare ventures to not only gain access to novel knowledge and skills, but also refine and extend their existing ones to cultivate ambidextrous capability. Accordingly, a coopetition strategy is especially crucial for digital healthcare ventures to facilitate ambidextrous capability when facing emerging opportunities and strong resource constraints. And these results also suggest that ambidextrous capability promotes growth performance by orchestrating connections between internally and externally available knowledge in digital healthcare ventures. Moreover, this research suggests that ambidextrous capability is the key mediator through which coopetition contributes to growth performance in digital healthcare ventures. To deal with high uncertainty and limited resources, they can use coopetition logic to engage in ambidextrous capability and thus develop growth performance successfully. However, with limited resources, the enterprise’s existing material and human resources will constrain the simultaneous development of exploration and exploitation capabilities. Given the differences in technical knowledge between the two, digital healthcare ventures must strike a balance between exploration and exploitation capabilities. Therefore, when ventures are pursuing long-term sustainable advantage, they focus more on the development of exploration capability. This study encourages them to proactively establish a cooperation network, assimilating external knowledge and skills through innovation alliances and collaborative R&D, learning new technologies, product development, management, and innovation skills, thereby enhancing their exploration capability and delivering better and more convenient digital healthcare services. Furthermore, when ventures expect to gain short-term profits from developing existing resources, they should focus on developing exploitation capability. They would choose to utilise coopetition to refine their knowledge and skills related to existing products, technologies, and user solutions, thereby boosting their exploitation capability to optimise and improve current healthcare services and increase service quality and patient satisfaction. In practice, ventures should focus on the construction of strategic management, organisational structure design, and employee management to ensure that they create ambidextrous capability.

Thirdly, our findings indicate that exploration capability is more useful for growth performance when digital healthcare ventures face higher environmental uncertainty. It is critical for digital healthcare ventures to recognise the impact of environmental uncertainty on business growth and clarify the direction of ambidextrous capability. To thrive, these ventures need to balance their exploration and exploitation capabilities under different environmental conditions, thereby enhancing organisational dynamic capabilities for successful change. Therefore, digital healthcare ventures should consciously take advantage of the dynamic development of ambidextrous capability and pay close attention to changes in the external environment so as to match ambidextrous capability with organisational development. In response to a dramatic environmental change, ventures should prioritise the acquisition of new knowledge and skills, innovating and introducing new products and services, and seeking potential opportunities in a dynamic environment. When facing relatively stable environments, ventures would rely more on experience or established routines to manage uncertainty and secure benefits.

### 5.4 Limitations and future research

This paper, while insightful, has certain limitations that pave the way for future research. Firstly, the focus of this study is on growth within digital healthcare ventures, and although the scales used were adapted from existing measures to align with the digital healthcare context, there’s a dearth of research-specific measurements for relevant variables within digital healthcare. Future research efforts will aim to develop more tailored measurement scales for digital healthcare through a synthesis of qualitative and quantitative research methods. Secondly, by targeting digital healthcare ventures, the study examines the mechanism of coopetition on growth performance. Comparative analyses with data from digitally mature firms could be helpful in future research to highlight distinctive patterns. Thirdly, this paper generalises the impact of coopetition on growth performance without delving into the effects of varying degrees of cooperation and competition. Further research could extend in this direction, perhaps by investigating the influence of coopetition intensity and diversity on growth performance in digital healthcare ventures. Fourthly, ambidexterity capability is considered as one dimension in this paper. Cao et al. (112) unpacked the one-dimensional ambidexterity construct into its “balance dimension” and its “combined dimension.” Raisch (116) argued that organisational ambidexterity can be achieved through differentiation or integration. These dimensions might rely on different causal mechanisms to enhance firm performance. This study advocates a more specific examination of the mechanisms at play in the different dimensions of organisational ambidexterity in future research. Lastly, this paper conducts a cross-sectional study, selecting digital healthcare ventures established within a relatively short timeframe. As has been observed by Lee and Roh (25), the coopetition strategy should be viewed from the perspective of a changing environment. A longitudinal approach could enable further research into the role of cooperation and competition as dynamic elements that transform growth performance, as well as how this helps firms exert their exploration and exploitation capabilities over time.

## Data availability statement

The raw data supporting the conclusions of this article will be made available by the authors, without undue reservation.

## Author contributions

XLi: Investigation, Methodology, Writing – original draft, Writing – review & editing. LB: Writing – review & editing. XLe: Writing – review & editing. YeY: Writing – review & editing. YuY: Writing – review & editing. DL: Writing – review & editing. HY: Funding acquisition, Investigation, Methodology, Writing – original draft, Writing – review & editing.
